# Sirt2 inhibition improves gut epithelial barrier integrity and protects mice from colitis

**DOI:** 10.1073/pnas.2319833121

**Published:** 2024-04-22

**Authors:** Dan Hou, Tao Yu, Xuan Lu, Jun Young Hong, Min Yang, Yanlin Zi, Thanh Tu Ho, Hening Lin

**Affiliations:** ^a^Department of Chemistry and Chemical Biology, Cornell University, Ithaca, NY 14853; ^b^HHMI, Cornell University, Ithaca, NY 14853; ^c^Department of Molecular Biology and Genetics, Cornell University, Ithaca, NY 14853

**Keywords:** inflammatory bowel disease, substrate-dependent inhibition, Arf6, sirtuins, E-cadherin

## Abstract

Sirt2 is a nicotinamide adenine dinucleotide (NAD^+^)-dependent protein lysine deacylase that can remove both acetyl group and long-chain fatty acyl groups from lysine residues of many proteins. It was reported to affect inflammatory bowel disease (IBD) symptoms in a mouse model. However, conflicting roles were reported, with genetic knockout aggravating while pharmacological inhibition alleviating IBD symptoms. These seemingly conflicting reports cause confusion and deter further efforts in developing Sirt2 inhibitors as a potential treatment strategy for IBD. We investigated these conflicting reports and elucidated the role of Sirt2 in the mouse model of IBD. We essentially replicated these conflicting results and confirmed that Sirt2 inhibitors’ protective effect is not through off-targets as two very different Sirt2 inhibitors (TM and AGK2) showed similar protection in the IBD mouse model. We believe that the differential effects of inhibitors and knockout are due to the fact that the Sirt2 inhibitors only inhibit some but not all the activities of Sirt2. This hypothesis is confirmed by the observation that a PROTAC degrader of Sirt2 did not protect mice in the IBD model, similar to *Sirt2* knockout. Our study provides an interesting example where genetic knockout and pharmacological inhibition do not align and emphasizes the importance of developing substrate-dependent inhibitors. Importantly, we showed that the effect of Sirt2 inhibition in IBD is through regulating the gut epithelium barrier by inhibiting Arf6-mediated endocytosis of E-cadherin, a protein important for the intestinal epithelial integrity. This mechanistic understanding further supports Sirt2 as a promising therapeutic target for treating IBD.

## Significance

Inflammatory bowel disease (IBD) is a chronic inflammatory disease of the gut that affects millions of people yearly. Understanding the disease mechanism and finding new therapeutic targets are important toward developing better therapeutics. Our study here establishes that small-molecule inhibitors of Sirt2 are promising treatment strategy for IBD. Small-molecule inhibitors of enzymes have been increasingly used to investigate the biological function of enzymes. Our study here provides an example where small-molecule inhibitors and genetic knockout do not produce the same phenotype in vivo, highlighting the distinct advantage of small-molecule inhibitors in achieving substrate-selective inhibition over genetic perturbation that affects all the activities of the enzymes.

Inflammatory bowel disease (IBD), including ulcerative colitis and Crohn’s disease, is a chronic inflammatory disease of the digestive tract (1). It affects millions of people each year and can be debilitating and cause life-threatening complications. While genome-wide association studies have identified many genetic variations that are associated with IBD (2–12), the exact cause of IBD remains unknown, making the development of molecular therapeutics difficult. While both small-molecule drugs and biologicals are available for treating IBD, there is no cure, and no treatment works on all patients (1). Thus, there is still a need for discovering new therapy for IBD treatment.

Sirt2 is a member of the sirtuin family of NAD^+^-dependent protein lysine deacylases. It can remove acetyl groups as well as long-chain fatty acyl groups from protein lysine residues (13). Sirt2 is known to work on many different substrate proteins (13), and its inhibition has been reported to offer beneficial effects in cancer and neurodegeneration (14–17). However, our understanding of its biological function is still fragmented, and in many cases, we still do not know how inhibiting Sirt2 produces such beneficial effects. Our interest in IBD drew our attention to two seemingly conflicting reports of Sirt2 in IBD (18, 19). Both reports concern the effects of Sirt2 in the dextran sodium sulfate (DSS)-induced colitis model in mice. In one report, *Sirt2* knockout mice performed worse than wild-type mice in DSS-induced colitis (19). However, in the other report, a Sirt2-specific small-molecule inhibitor, TM, protected mice in DSS-induced colitis (18). These reports seemingly contradict each other. In both cases, no convincing molecular mechanisms were available to explain the reported effect of Sirt2 in colitis.

We were curious to find out whether Sirt2 inhibition/deletion offers protection in DSS-induced colitis and attempted to validate the literature experiments and further investigate the molecular mechanism for the role of Sirt2 in DSS-induced colitis.

## Results

### Sirt2 Inhibition Protects Mice in DSS-Induced Colitis.

**Fig. 1.**
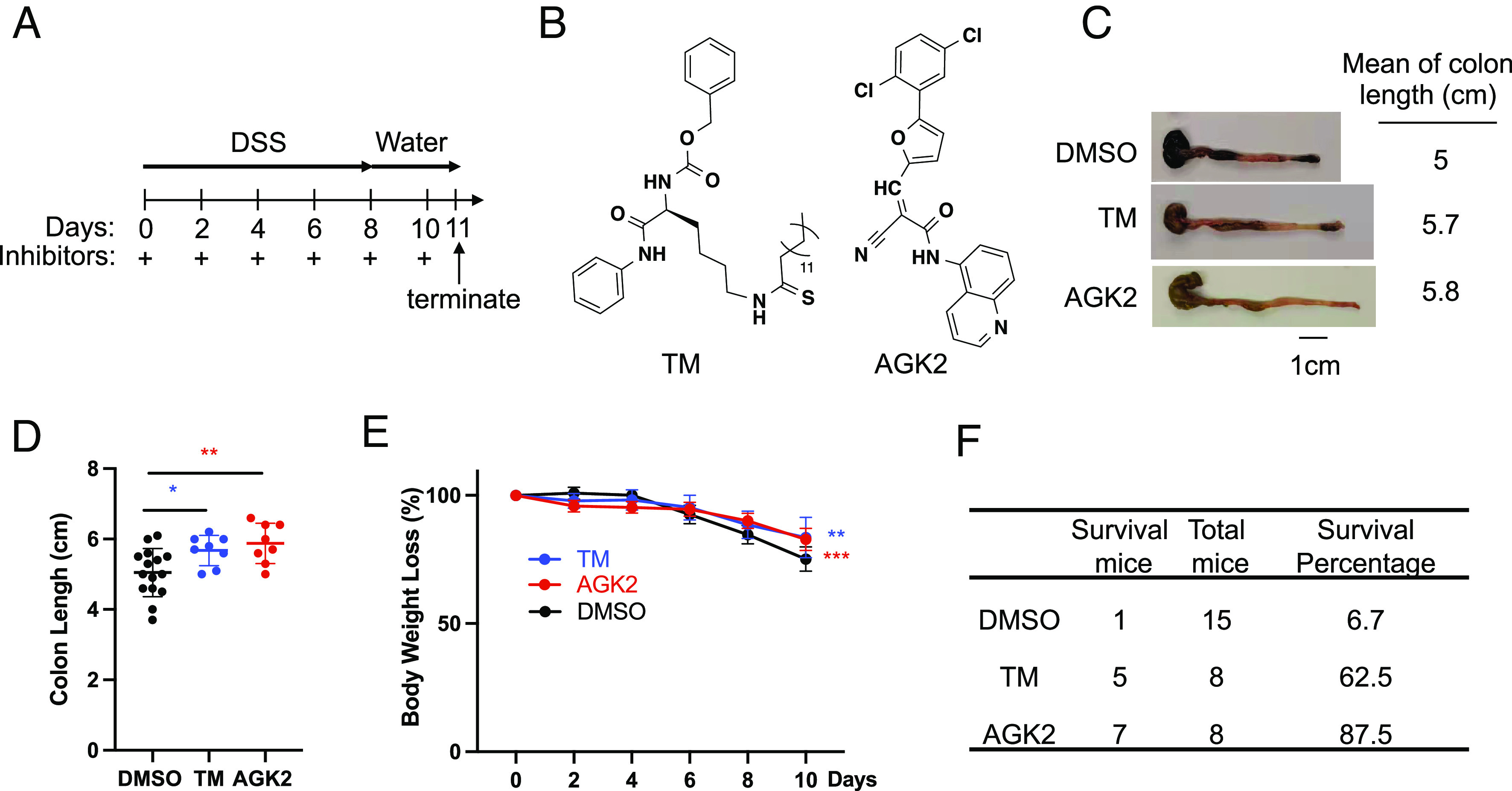
Sirt2 inhibition protects mice in DSS-induced colitis. (*A*) The timeline showing the treatment schedule in the 2.5% DSS colitis model in C57BL/6 mice. (*B*) Structures of Sirt2 inhibitors: TM (thiomyristoyl-lysine) and AGK2. (*C*) Sirt2 inhibitors TM and AGK2 protected C57BL/6 mice from colon shortening induced by DSS. (*D*) Quantitative analysis of colon length in different mouse groups treated with DSS. (*E*) Body weight loss of different mouse groups treated with DSS. (*F*) Survival rate of different mouse groups treated with DSS. ***P < 0.001, *P < 0.05, and **P < 0.01 (Student’s *t* test). Error bars: ±SD.

To explore the effects of Sirt2 inhibitors in the colitis mouse model, we administered 8 to 12-wk C57BL/6 mice with 2.5% DSS to induce colitis. For Sirt2 inhibitors, we chose two very different ones to make sure that the pharmacological effect observed was not due to off-target effects. One Sirt2 inhibitor is a mechanism-based thiomyristoyl-lysine compound, TM, which was developed by our laboratory (16). The other Sirt2 inhibitor is AGK2, which is a substrate competitive inhibitor developed by others and widely used by many labs (14). Mice were treated following the timeline shown in Fig. 1 *A* and *B*. We found that compared to the DMSO control group, both TM and AGK2 inhibited the reduction of colon length induced by DSS (Fig. 1 *C* and *D*). Moreover, the TM and AGK2 group showed improvement of body weight loss and survival rate (Fig. 1 *E* and *F*). Thus, we were able to replicate the previous report that Sirt2 inhibitor TM protects mice in the DSS-induced colitis model. Furthermore, given that similar effects were observed with a very different inhibitor, AGK2, we can rule out that the protective effect is due to off-target effect and conclude that Sirt2 inhibition protected mice in DSS-induced colitis.

### *Sirt2* Knockout Does Not Protect Mice in DSS-Induced Colitis.

**Fig. 2.**
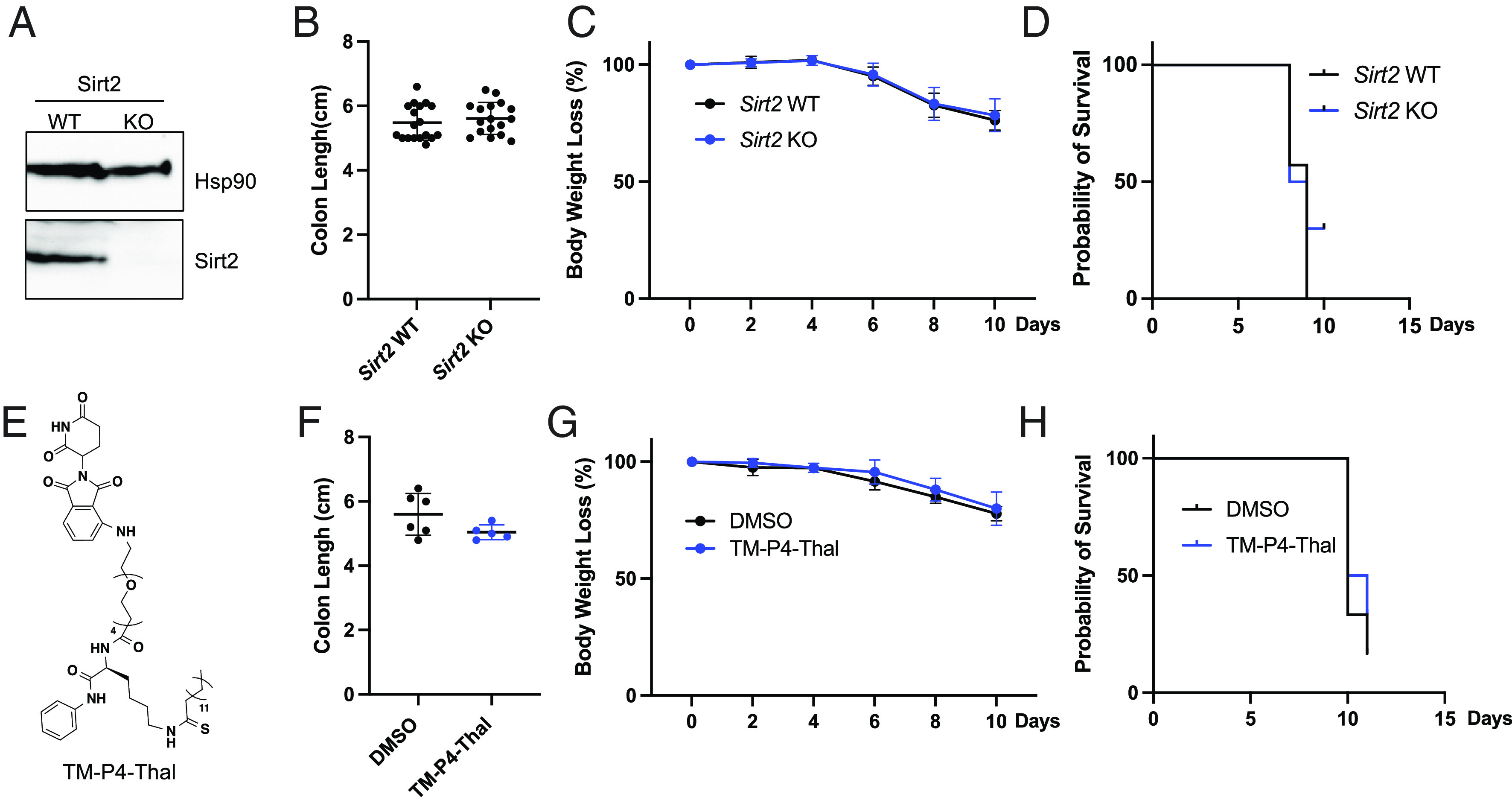
*Sirt2* knockout or PROTAC degrader does not protect mice in DSS-induced colitis. (*A*) Immunoblots of Sirt2 in spleen cells from 8-wk *Sirt2* wild-type and knockout mice. (*B*) Colon length of *Sirt2*+/+ and *Sirt2*–/– mice treated with 2.5% DSS. (*C*) Body weight of *Sirt2*+/+ and *Sirt2*–/– mice treated with 2.5% DSS. (*D*) Survival probability of *Sirt2*+/+ and *Sirt2*–/– mice. (*E*) Structures of TM-P4-Thal. (*F*) Colon length of mice treated with DMSO and TM-P4-Thal in the DSS model. (*G*) Body weight of mice treated with DMSO and TM-P4-Thal in the DSS model. (*H*) Survival probability of mice treated with DMSO and TM-P4-Thal in the DSS model. Error bars: ±SD.

It was previously reported that *Sirt2* knockout mice are slightly more susceptible to DSS-induced colitis, which is opposite to the *Sirt2* inhibition phenotype. To validate this report, we administrated 8 to 12-wk *Sirt2*+/+ and *Sirt2*−/− age-matched and littermate mice (Fig. 2*A*) with 2.5% DSS to induce colitis. Unlike Sirt2 inhibitors, *Sirt2* knockout did not offer protection in mice with DSS-induced colitis, as evidenced by the similar colon lengths, body weights, and survival rates in *Sirt2*+/+ and *Sirt2*−/− mice (Fig. 2 *B*–*D*).

The phenotype of the *Sirt2* knockout was clearly different from that of the Sirt2 inhibitors. At the same time, we were confident that the Sirt2 inhibitor phenotype was reliable as we used two very different inhibitors to rule out off-target effects of small-molecule inhibitors. Given these observations and considerations, how can we rationalize the different effects of Sirt2 inhibition and knockout?

Sirt2 has multiple catalytic activities. It can remove acetyl groups from multiple proteins, such as HIF1α (20), LDH-A (21), and GSK3β (22). At the same time, it can also efficiently remove long-chain fatty acyl groups from small GTPases KRas4a (23), RalB (24), and Arf6 (25). Interestingly, it has been demonstrated that most Sirt2 inhibitors, including TM and AGK2, can inhibit the deacetylation activity of Sirt2 very well but cannot inhibit the defatty-acylation activity of Sirt2 very well (26, 27). This is likely because fatty-acyl lysine substrates can bind Sirt2 more tightly and thus can effectively compete with the small-molecule inhibitors, while the acetyl lysine substrate binds Sirt2 less tightly and cannot compete with the inhibitors. However, the situation is slightly more complex as we have demonstrated that while TM cannot inhibit Sirt2-catalyzed defatty-acylation of KRAS4A (28), it can inhibit Sirt2-catalyzed Arf6 demyristoylation (25). Thus, many small-molecule inhibitors of Sirt2, including TM and AGK2, affect Sirt2’s function in a substrate-dependent manner and only disrupt its function on a subset of its substrate proteins. In contrast, Sirt2 knockout would affect all its substrate proteins, providing a possible explanation for the different effect of *Sirt2* knockout from that of Sirt2 inhibition in the DSS-induced colitis model.

To provide experimental support for this hypothesis, we decided to use a PROTAC inhibitor of Sirt2, TM-P4-Thal (Fig. 2*E*), which was previously developed in our lab (29). TM-P4-Thal is a bifunctional molecule that can bind Sirt2 with the TM portion and recruit the CRBN E3 ubiquitin ligase with the thalidomide portion to ubiquitinate Sirt2, leading to Sirt2 degradation and thus inhibition of all of its activities (29). Therefore, if our hypothesis above is correct, we expect that TM-P4-Thal would have similar effect as *Sirt2* knockout in mice in the DSS-induced colitis model. As expected, treatment with TM-P4-Thal resulted in slightly shorter (but not statistically significant) colon length and no benefit in survival rate and body weight loss (Fig. 2 *F*–*H*), which is similar to *Sirt2* knockout and different from TM and AGK2 treatment. These results support our hypothesis that Sirt2 partial or substrate-dependent inhibition is important for providing beneficial effects in the DSS-induced colitis model.

### Sirt2 Inhibition Increases Surface E-cadherin through Suppressing Arf6 Activation.

We next sought to understand the mechanism via which Sirt2 affects DSS-induced colitis. Previous genome-wide association studies identified *C1orf106* mutation in humans to be associated with increased risk of inflammatory bowel diseases (10–12). Interestingly, *C1orf106* encodes a protein that negatively regulates the guanine nucleotide exchange factor (GEF) for Arf6 (30). IBD-associated *C1orf106* mutations increases Arf6 activation (GTP bound Arf6, Arf6-GTP), which in turn impairs the integrity of intestinal epithelial cells by promoting the endocytosis of E-cadherin, a protein that is important for maintaining the epithelial barrier (30). Our previous work revealed that ARF6 is regulated by a myristoylation–demyristoylation cycle on Lys3 that is catalyzed by N-terminal myristoyltransferase (NMT) and SIRT2 (25). This myristoylation–demyristoylation cycle promotes the GTPase cycle of ARF6, and thus, inhibiting either NMT or SIRT2 leads to the disruption of this myristoylation–demyristoylation cycle and inhibition of ARF6 activation (25). We therefore hypothesized that Sirt2 is involved in colitis by regulating Arf6 fatty-acylation and activation, thus influencing the integrity of intestinal epithelium.

To investigate this hypothesis, we first examined whether SIRT2 disruption decreased ARF6 activation in Caco2 cells, a human colon cell line. As expected, SIRT2 knockdown or inhibition decreased the ARF6-GTP level (*SI Appendix*, Fig. S1 *A*–*C*). Similar effect was also observed in HEK 293T cells (*SI Appendix*, Fig. S1 *D* and *E*). We next tested whether SIRT2 disruption inhibited the endocytosis of E-cadherin and increased the cell surface level of E-cadherin. We monitored the surface E-cadherin by biotinylating Caco2 extracellular membrane proteins, pulling down the biotinylated proteins, and immunoblotting for E-cadherin. As predicted, SIRT2 knockdown or inhibition increased cell surface E-cadherin (*SI Appendix*, Fig. S1 *F* and *G*). The subcellular fractionation experiment further supports that SIRT2 disruption increased plasma membrane localization of E-cadherin (*SI Appendix*, Fig. S1 *H* and *I*).

**Fig. 3.**
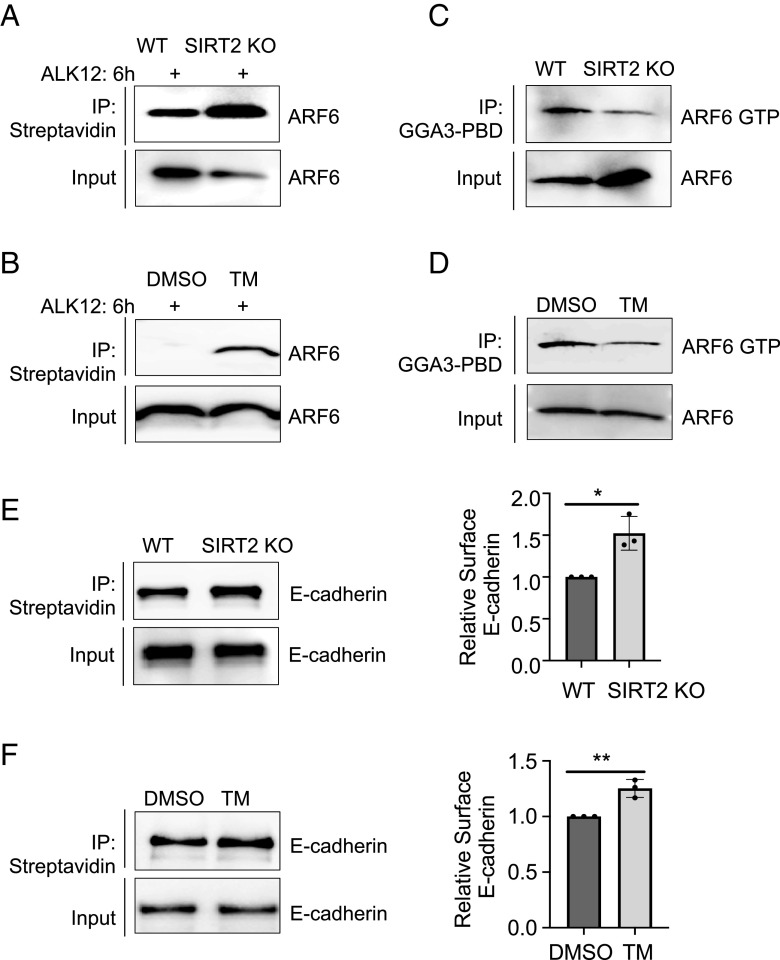
*Sirt2* deficiency increases surface E-cadherin by suppressing ARF6 activation. (*A*) The fatty acylation level of Arf6 in mouse primary colon cells obtained from *Sirt2* WT and KO mice treated with DSS. (*B*) The fatty acylation level of Arf6 in mouse primary colon cells obtained from mice treated with DSS and DMSO or TM (50 mg/kg). (*C*) The levels of activated Arf6 (Arf6-GTP) in mouse primary colon cells obtained from *Sirt2* WT and KO mice treated with DSS. Arf6-GTP was immunoprecipitated using the GGA3-PBD agarose beads. The protein binding domain (PBD) of GGA3 (Golgi-localized, gamma ear-containing, ARF-binding protein) binds Arf6-GTP selectively and allows the pull down of Arf6-GTP. (*D*) The levels of activated Arf6 (Arf6-GTP) in mouse primary colon cells obtained from DSS-induced colitis mice treated with DMSO or TM. Arf6-GTP was immunoprecipitated using the GGA3-PBD agarose beads. (*E*) Surface levels of E-cadherin in mouse primary colon cells obtained from *Sirt2* WT and *Sirt2* KO mice treated with DSS. Surface levels of E-cadherin were determined using cell surface protein biotinylation, streptavidin pull down, and western blotting (*Left*). The ratio of surface to total E-cadherin was quantified and shown as a bar graph (*Right*). (*F*) Surface levels of E-cadherin in mouse primary colon cells obtained from DSS-induced colitis mice treated with DMSO or TM. Surface levels of E-cadherin were determined by western blotting (*Left*). The ratio of surface to total E-cadherin was quantified and shown as a bar graph (*Right*).*P < 0.05 and **P < 0.01 (Student’s t test). Error bars: ±SD.

In a more physical context, we determined Arf6 activation and E-cadherin endocytosis in primary epithelial colon cells from DSS-induced colitis mice. As expected, *Sirt2* knockout or Sirt2 inhibition increased the fatty-acylation level of Arf6 (Fig. 3 *A* and *B*), decreased Arf6-GTP level (Fig. 3 *C* and *D*), and increased cell surface E-cadherin (Fig. 3 *E* and *F*).

Taken together, inhibition of Sirt2 suppresses Arf6 activation, which in turn inhibits the endocytosis of E-cadherin and promotes the cell surface level of E-cadherin.

### Suppressing Arf6 Activation by an Nmt Inhibitor Offers a Protective Role in Mice with DSS-Induced Colitis.

**Fig. 4.**
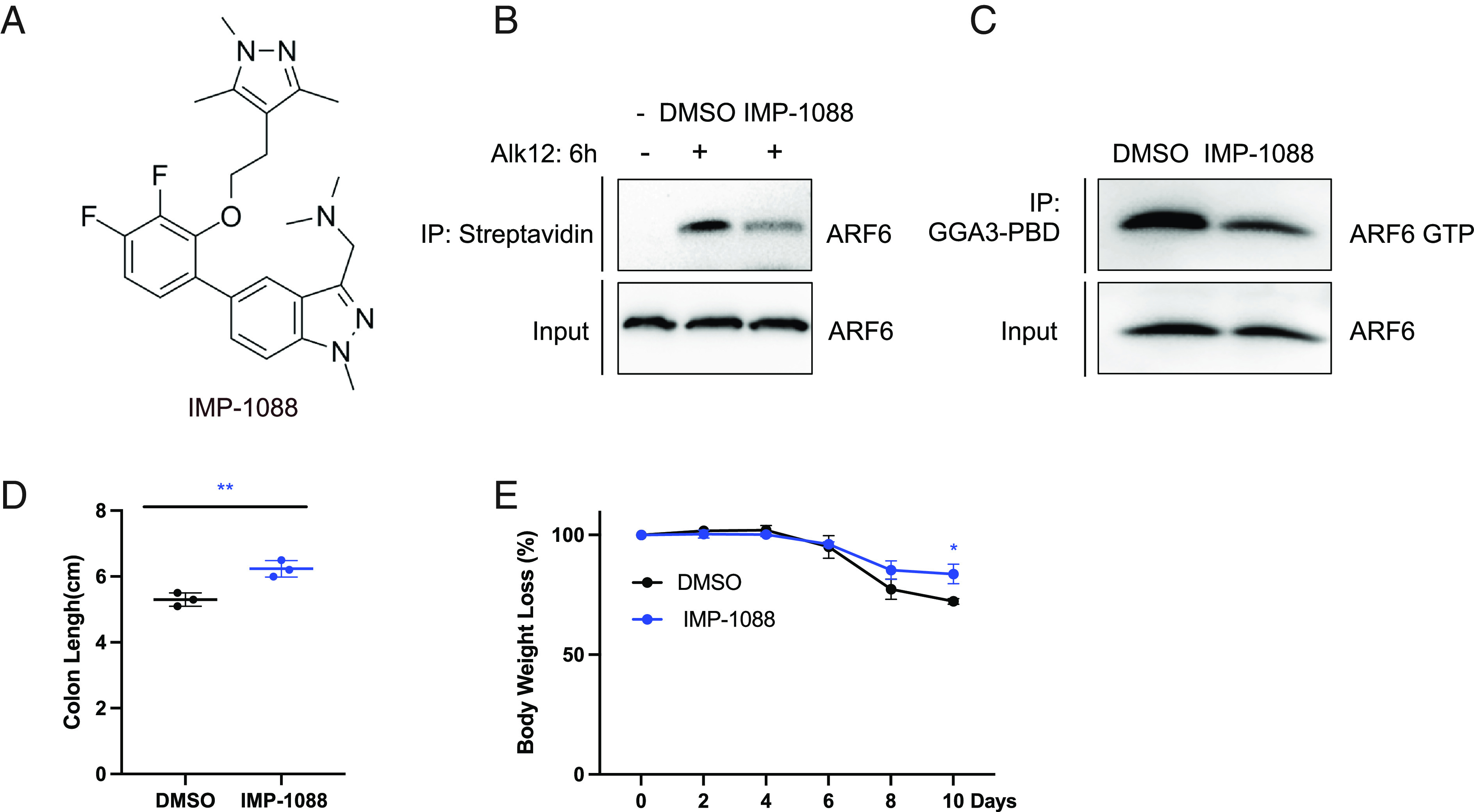
Suppressing Arf6 activation with an Nmt inhibitor offers a protective role in mice with DSS-induced colitis. (*A*) Structures of Nmt inhibitor IMP-1088. (*B*) The fatty acylation level of ARF6 in Caco2 cells treated with DMSO and 0.1 μM IMP-1088 for 24 h. (*C*) The levels of activated ARF6 (ARF6-GTP) in Caco2 cells treated with DMSO and 0.1 μM IMP-1088 for 24 h. ARF6-GTP was immunoprecipitated using the GGA3-PBD agarose beads. (*D*) Quantitative analysis of colon length in DSS-induced colitis mice treated with DMSO or Nmt inhibitors IMP-1088 (5 mg/kg). (*E*) Body weight loss of DSS-induced colitis mice treated with DMSO or IMP-1088 (5 mg/kg). *P < 0.05 and **P < 0.01 (Student’s *t* test). Error bars: ±SD.

The myristoylation–demyristoylation cycle promotes the GTPase cycle of ARF6, and thus, inhibiting either NMT or SIRT2 leads to the disruption of this cycle and inhibition of ARF6 activation (25). We therefore hypothesized that Nmt inhibitor should also protect mice from DSS-induced colitis. To validate this hypothesis, we used a picomolar Nmt inhibitor, IMP-1088 (Fig. 4*A*). In Caco2 cells, IMP-1088 decreased the fatty-acylation level of the ARF6 and ARF6-GTP level (Fig. 4 *B* and *C*), just like the myristoylation–demyristoylation cycle predicted. IMP-1088 also offered protection in the DSS-induced colitis mouse model, leading to longer colon length (Fig. 4*D*) and less body weight loss (Fig. 4*E*). These results further support that the Nmt and Sirt2-catalyzed myristoylation–demyristoylation cycle of Arf6 plays an important role in DSS-induced colitis.

### Sirt2 Deficiency Improves Epithelial Barrier Integrity.

**Fig. 5.**
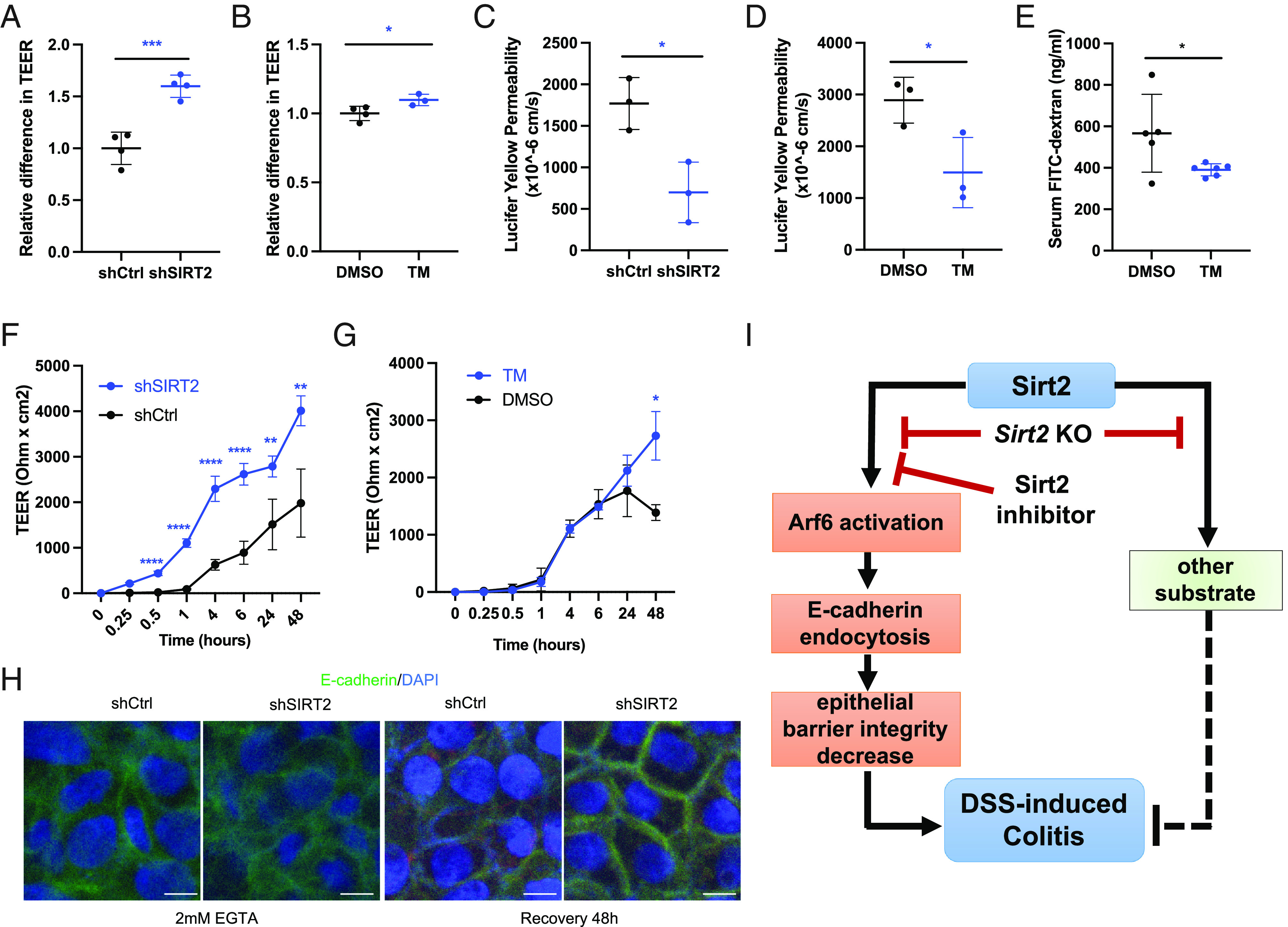
Inhibition of Sirt2 increases epithelial barrier integrity. (*A*) Relative maximal TEER in control and SIRT2 knockdown Caco2 cells. (*B*) Maximal TEER in Caco2 cells treated with DMSO or 25 μM TM. (*C*) Lucifer Yellow permeability in control and SIRT2 knockdown Caco2 cells. (*D*) Lucifer Yellow permeability assay in Caco2 cells treated with DMSO or 25 μM TM. (*E*) Serum FITC-dextran permeability in DSS-induced colitis mice treated with DMSO or TM (50 mg/kg). (*F*) TEER measurement at various time points after calcium switch treatment (2 mM EGTA) in control and SIRT2 knockdown Caco2 cells. (*G*) TEER measurement at various time points after calcium switch treatment in Caco2 cells treated with DMSO or 25 μM TM. (*H*) Confocal images of control and SIRT2 knockdown Caco2 cells treated with 2 mM EGTA for 8 min. After EGTA treatment, cells were allowed to recover for 48 h. Cells were stained for E-cadherin (green) and nuclei (blue). (Scale bar: 10 μm.) (*I*) A model for the regulation of DSS-induced colitis via Sirt2 inhibition. ****P < 0.0001, ***P < 0.001, *P < 0.05, and **P < 0.01 (Student’s *t* test). Error bars: ±SD.

E-cadherin recycling plays an important role in epithelial junction integrity and regulates the permeability of epithelial cells in the colitis model. Therefore, we next tested whether Sirt2 inhibition or knockdown would improve the epithelial barrier integrity. Caco2 cell is widely used as an epithelial cell model; therefore, we first tested the permeability of Caco2-formed monolayer cells by transepithelial electrical resistance (TEER) (30). Maximal TEER was significantly increased in SIRT2 knockdown and inhibition groups compared with the control (Fig. 5 *A* and *B*), suggesting improved epithelial barrier integrity with Sirt2 knockdown or inhibition. Next, we tested the ability of fluorescently labeled molecules Lucifer Yellow to pass through the Caco2 monolayer cells (31). SIRT2-deficient or inhibition in Caco2 cells exhibited reduced permeability to Lucifer Yellow (Fig. 5 *C* and *D*). Finally, we tested the ability of FITC-dextran (4 kDa) to pass the intestinal barrier in mice (30). Sirt2 inhibition in mice showed reduced permeability to FITC-dextran (*SI Appendix*, Fig. S2). Importantly, in DSS-induced colitis mice, Sirt2 inhibitor treatment also reduced mouse gut permeability to FITC-dextran (Fig. 5*E*). Thus, these results indicated that inhibition of Sirt2 improved epithelial barrier integrity.

To further connect the effect of SIRT2 in epithelial barrier integrity regulation with ARF6, we overexpressed ARF6 in WT and SIRT2 knockdown Caco2 cells (*SI Appendix*, Fig. S3*A*) and detected the epithelial barrier integrity using TEER assay. We found that SIRT2 knockdown improved the epithelial barrier integrity, while ARF6 overexpression could counteract the impact of SIRT2 knockdown (*SI Appendix*, Fig. S3*B*), further implying the SIRT2-ARF6 axis in the regulation of epithelial barrier integrity.

To further confirm these findings, we investigated the ability of Caco2 cells to repair the epithelial junctions after injury using a calcium switch assay (30). EGTA (2 mM) was added to the Caco2 monolayer cells to disrupt the epithelial junctions, and 8 min later, the media were switched back to normal media to allow for recovery. We then performed TEER during this recovery phase. Compared with the control groups, SIRT2 knockdown or inhibition increased the TEER, suggesting better recovery from repair with SIRT2 knockdown or inhibition (Fig. 5 *F* and *G*). EGTA also disrupts extracellular E-cadherin junction (32), and we also tested whether the E-cadherin junctional localization was restored during the recovery phase. Immunofluorescence of E-cadherin showed that SIRT2 knockdown improved E-cadherin junction localization, suggesting that SIRT2 could repress E-cadherin junctional localization, which was critical for epithelial barrier integrity in this case (Fig. 5*H*).

Overall, our data support a model that Sirt2 inhibition provides beneficial effects in the DSS-induced colitis model with a substrate-dependent mechanism. Inhibition of Sirt2 will promote the epithelial barrier integrity via inhibiting Arf6-catalyzed E-cadherin endocytosis (Fig. 5*I*). The differential effects of small-molecule inhibitors and genetic knockout are due to the fact that the Sirt2 inhibitors only inhibit some but not all the activities of Sirt2, while the knockout affects all the activities of Sirt2.

## Discussion

Our study here validated two seemingly conflicting reports regarding the role of Sirt2 in the DSS-induced mouse colitis model. We confirmed that *Sirt2* genetic knockout does not provide protection, while small-molecule inhibitors of Sirt2 offer clear protection in the DSS colitis model. In addition, we ruled out that the protective effects of small-molecule inhibitors are due to off-target effect as two very different inhibitors developed by different labs both showed similar protective effects. Furthermore, a PROTAC Sirt2 degrader also produced a phenotype similar to that of *Sirt2* knockout.

While in general, genetic and pharmacological perturbation produce consistent phenotypes, there have been examples in the literature that the two approaches can produce different phenotypes (33). This has been generally attributed to the fact that an enzyme could have both catalytic activities and scaffolding roles, and genetic perturbation gets rid of both, while small-molecule inhibitors only get rid of the catalytic activity (33). In the Sirt2 case, we believe that the reason that small-molecule inhibitors produce a different phenotype is because the small-molecule inhibitors’ effect is substrate dependent as they only inhibit Sirt2’s activity on certain, but not all, substrates. For example, it has been previously shown that TM can inhibit the deacetylation activity of Sirt2 as well as the demyristoylation of Arf6 (16, 25), but it cannot inhibit the deacylation of KRas4a (28). This has also been shown by several other Sirt2 inhibitors (26, 27). This was verified in our system that SIRT2 inhibitor TM only impacted the fatty acylation level on ARF6 but not KRAS4a in Caco2 cells (*SI Appendix*, Fig. S4). Such substrate-dependent inhibitors may produce effects that are different from the complete knockout of the enzyme (34). Thus, they present an exciting opportunity for potential clinical applications.

In addition to establishing that Sirt2 inhibition and knockout produce different phenotypes, our work also provides an interesting molecular mechanism for how Sirt2 disruption protects mice in the DSS-induced colitis model (Fig. 5*I*). This is connected to a previously established Sirt2 demyristoylation substrate, Arf6. Arf6 is a small GTPase important for the endocytosis of certain cell surface proteins (35, 36), including E-cadherin (30), which is important for the intestinal epithelium integrity. By inhibiting Sirt2, the endocytosis of E-cadherin is inhibited, leading to an improved epithelium barrier and protection of mice in the colitis model.

Interestingly, the Arf6 pathway has been previously associated with IBD (30). Genome-wide association studies have identified *C1orf106* mutations to be associated with increased IBD risk (10–12). C1orf106 is an inhibitor for the GEF of Arf6 and the IBD-associated mutation disrupts C1orf106, leading to increased Arf6 activation and risk of colitis (30). Our study further established the importance of Arf6 pathway in IBD and offered Sirt2 as unique target to regulate Arf6 and treat IBD. Since previous studies showed that Sirt2 inhibitors are well tolerated in mice and Sirt2 knockout mice has only a very mild phenotype (16, 19, 37), targeting Sirt2 can be a safe and promising strategy to treat IBD.

## Methods

### Plasmids, Reagents, and Antibodies.

hARF6 ORF cDNA lentiviral clone was obtained from GeneCopoeia (EX-OL00097-LX304-B).

DSS was from Cayman Chemical. Puromycin, hexadimethrine bromide (polybrene), Lucifer Yellow CH dilithium salt, and fluorescein isothiocyanate–dextran were purchased from Sigma. EZ-Link Sulfo-NHS-Biotin and Collagenase, Type I, were obtained from Thermo Fisher Scientific. Polyethylenimine (PEI) was from Polysciences. AGK2 was from MCE. TM, TM-P4-Thal, and IMP-1088 were synthesized as previously described (29, 38, 39).

Antibodies were obtained from the following sources: Cell Signaling Technology—rabbit anti-E-cadherin (24E10), E-cadherin (24E10) rabbit mAb (Alexa Fluor® 488 Conjugate), rabbit anti-SirT2 (D4050), rabbit anti-HSP90 (C45G5), rabbit anti-Arf6 (D12G6), anti-mouse IgG, HRP-linked antibody (#7076), anti-rabbit IgG, and HRP-linked antibody (#7074); Santa Cruz—anti-β-actin antibody (C4) and anti-GAPDH (0411).

### Cell Culture and Transfection.

Caco2 cells were cultured in Eagle’s minimum essential medium (EMEM, ATCC) supplemented with 20% (vol/vol) fetal bovine serum (FBS; Invitrogen). HEK 293T cells were grown in Dulbecco’s Modified Eagle Medium (DMEM, Invitrogen) supplemented with 10% (vol/vol) FBS. Cells were incubated in a humidified chamber at 37 °C with 5% CO2. SIRT2 stable knockdown HEK 293T and Caco2 cells were generated using the methods described previously (26).

### Mice.

C57BL/6 and *Sirt2* knockout (stock #012772) mice were purchased from JAX lab. All experiments involving mice followed the protocols approved by the Institutional Animal Care and Use Committee of Cornell University.

### DSS-Induced Colitis.

Mice (8 to 12 wk) were provided with 2.5% (w/v) DSS dissolved in sterile water as drinking water for 8 d and then changed to normal drinking water without DSS for 3 d. Mice were killed if the body weight loss was more than 20%. Animal weights were monitored every other day. Mice were killed on day 11, and colons were collected to measure the colon length. For the inhibitor treatment experiments, we treated the mice with 50 mg/kg TM, 50 mg/kg TM-P4-Thal, 5 mg/kg IMP-1088, and the same volume DMSO as control. The inhibitors were treated on days 0, 2, 4, 6, 8, and 10.

### ARF6 Activity Assay.

The ARF6 activity assay was detected according to a kit (BK033) from Cytoskeleton following the kit’s protocol and previously published procedure (30).

### Calcium Chelation Assay.

Caco2 cells were seeded on polytetrafluoroethylene filters (Transwell, Corning, 24 mm Transwell® with 0.4 μm Pore Polyester Membrane Insert, Sterile) for 21 d to form a monolayer. The monolayer was treated with 2 mM EGTA for 8 min and then switched to fresh media. The cells were then examined by confocal imaging for E-cadherin localization or by TEER for electrical resistance.

### Confocal Immunofluorescence.

Caco2 cells were plated on polytetrafluoroethylene filters (Transwell, Corning, 24 mm Transwell® with 0.4 μm Pore Polyester Membrane Insert, Sterile) for 21 d. Cells were washed with PBS, fixed in freshly made 4% (w/v) paraformaldehyde (15 min, 25 °C), permeabilized with 0.1% Triton X-100 (v/v), and then blocked in 3% (w/v) bovine serum albumin in PBS for 30 min. Cells were labeled with anti-E-cadherin (1:200) Alexa Fluor® 488–conjugated antibody overnight at 4 °C. The cells were washed three times with PBS and then stained with DAPI. Images were acquired with a Zeiss 710 confocal microscope using a 40× objective and presented with 4× Crop. The ZEN software was used to process images.

### TEER.

A total of 1 × 10^4^/well Caco2 cells were seeded and grown for 21 d to form monolayers on polytetrafluoroethylene filters (Transwell, Corning, 24 mm Transwell® with 0.4 μm Pore Polyester Membrane Insert, Sterile) with medium changes every 2 d. TEER was measured using chopstick electrodes and Millicell ERS-2 Voltohmmeter (Millipore) daily until maximal TEER was attained. The relative resistance was then calculated.

### Biotinylation of Cell Surface E-cadherin and Immunoprecipitation.

The Pierce™ Cell Surface Biotinylation and Isolation Kit (Thermo Fisher Scientific) was used for detecting the cell surface proteins. In brief, Caco2 cells were washed with ice-cold PBS and incubated in 2 mM biotin solution in PBS at 4 °C for 30 min. The cells were then washed twice with the supplied TBS buffer. Then, the cells were lysed with 500 μL supplied lysis buffer with protease inhibitors for 30 min at 4 °C on a rotator. To perform immunoprecipitation of biotinylated E-cadherin, after taking out 40 μL as input samples, the rest of the supernatant of cell lysate was incubated with 250 μL streptavidin beads for 30 min at room temperature. The beads were washed three times with 500 μL supplied wash buffer and then mixed with 70 μL of protein loading buffer and boiled for 10 min. The samples were then resolved by SDS-PAGE and analyzed by western blot for E-cadherin.

### Western Blot Analysis.

Western blots were performed as described previously (26). The proteins were detected using enzyme-linked fluorescence (Clarity Max, Bio-Rad; ECL Plus, Pierce Biotechnology Inc.) and visualized using the ChemiDoc (Bio-Rad).

### FITC-Dextran Mouse Intestinal Permeability Assay.

C57BL/6 mice (8 to 12 wk old, both males and females) were used for the experiments and injected every other day with 50 mg/kg TM or DMSO control three times via intraperitoneal (IP) injection. After the last Sirt2 inhibitor injection on day 5, mice were fasted for 4 to 6 h prior to the administration of FITC-dextran. FITC-dextran (4KDa) was dissolved in sterile 1× PBS at 44 mg/400 μL. Mice were gauged with 44 mg FITC dextran/100 g body immediately after fasting. Blood was collected 4 h postgavage in anticoagulation tubes, mixed thoroughly by inversion, and kept on ice before centrifugation at 3,000 g for 5 min and transferring the plasma to a new 1.5 mL microfuge tube. Then, 100 μL of the plasma was transferred to a black opaque flat-bottom 96-well plate, and the fluorescence was determined at 528 nm with excitation at 485 nm. Permeability is expressed as relative fluorescence units between the groups being compared.

### Caco2 Cell Lucifer Yellow Permeability Assay.

Caco2 cells were placed on polytetrafluoroethylene filters (Transwell, Corning) and cultured for 21 d to form monolayer cells. The top chamber was washed with phenol red-free DMEM and then 100 μL of 1 mM Lucifer Yellow dissolved in phenol red-free DMEM were added. A volume of 600 μL phenol red-free DMEM was added on the lower chamber. Then, 100 μL of DMEM was collected from the lower chamber, and Lucifer Yellow levels were determined at an excitation wavelength of 428 nm by a microplate reader. Permeability was calculated using the following formula: P_app_ (cm/s) = (dQ/dt)/(A × C_0_ × 60). dQ/dt is the amount of substance transported per min (ng/min or RFU), A is the surface area of filter (cm^2^), C_0_ is starting concentration of substance (ng/mL), and 60 is the conversion of min to sec (31).

### Fractionation Assay.

Following the protocol provided by Minute™ Plasma Membrane Protein Isolation and Cell Fractionation Kit (Invent Biotechnology/Fisher), 293T cells were harvested and performed fractionation for the plasma membrane. The cells were washed once with cold PBS. The cells were resuspended in 500 μL buffer A and incubated on ice for 5 to 10 min. The suspension was vigorously vortexed for 10 to 30 s and then immediately transferred to a filter cartridge. The filter cartridge was capped and centrifuged at 14,000 rpm for 1 min. A volume of 40 μL of the lysate was taken out as input; the rest was centrifuged at 3,000 rpm for 1 min to collect the supernatant. The supernatant was centrifuged at 16,000 g for 30 min to collect the pellets. To the pellet, 200 μL of buffer B plus protease inhibitors were added. The pellet was resuspended and centrifuged at 16,000 g for 30 min. Then, 30 μL loading buffer was added to the pellets and boiled for 10 min before SDS-PAGE and western blot analysis.

### Primary Epithelial Colon Cell Isolation.

Primary epithelial colon cells were isolated as previously described (40). Briefly, colonic tissue was cutted into small pieces and then incubated in collagenase digestion for 60 to 90 min at 37 °C, followed by manual disruption of the tissue by pipetting.

### Lentiviral Particle Production and Transduction of Caco-2 Cells.

hARF6 ORF cDNA lentiviral clone was obtained from GeneCopoeia (EX-OL00097-LX304-B). This vector was mixed with psPAX2 and pMD2.G in the ratio 5:3:2 and transfected in HEK293T cells with PEI. After 48 h transfection, lentivirus-containing medium was collected and filtered through a 0.45 μm filter. The virus was used to infect the Caco2 cells with 8 μg/mL polybrene and media were changed 12 h later. After 48 h of infection, cells were selected in 10 μg/mL puromycin for 3 d.

### Statistical Analysis.

The Student’s *t* test was used to analyze data. *P < 0.05, **P < 0.01, ***P < 0.001, and ****P < 0.0001 represent the significance. Error bars: ±SD.

## Supplementary Material

Appendix 01 (PDF)

## Data Availability

All study data are included in the article and/or *SI Appendix*.
