# Supplementary material for: Sirt2 inhibition improves gut epithelial barrier integrity and protects mice from colitis
Source: Proc Natl Acad Sci U S A. 2024 Apr 22;121(18):e2319833121. doi: 10.1073/pnas.2319833121 (PMC11066986; doi:10.1073/pnas.2319833121)
Supplement: Supplementary file 1 — Appendix 01 (PDF) [file pnas.2319833121.sapp.pdf]

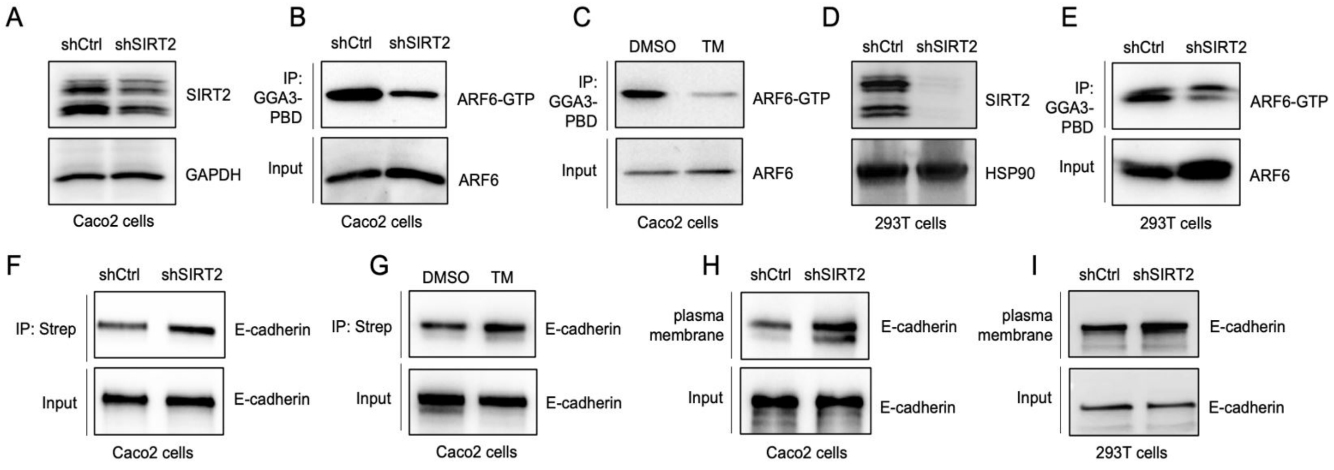

Figure S1. SIRT2 inhibition increases surface E-cadherin through suppressing ARF6 activation. (A) SIRT2 protein levels in Caco2 cells with control or SIRT2 knockdown. **(**B) The levels of activated ARF6 (ARF6-GTP) in control and SIRT2 knockdown Caco2 cells. ARF6-GTP was immunoprecipitated using the GGA3-PBD agarose beads. (C) The levels of activated ARF6 in Caco2 cells treated with DMSO or TM, determined using GGA3-PDB pull down. (D) SIRT2 protein levels in HEK293T cells with control of SIRT2 knockdown. (E) The levels of activated ARF6 (ARF6-GTP) in control and SIRT2 knockdown HEK293T cells. (F) Surface levels of E-cadherin in control or SIRT2 knockdown Caco2 cells. (G) Surface levels of E-cadherin in Caco2 cells treated with DMSO or TM. Surface levels of E-cadherin in F and G were determined using cell surface protein biotinylation, streptavidin pull down, and western blotting. (H-I) Plasma membrane fraction of E-cadherin in WT and SIRT2 knockdown Caco2 cells (H) or HEK 293T cells (I). The total cell lysates were fractionated to obtain the plasma membrane fractions, which were then blotted for E-cadherin.

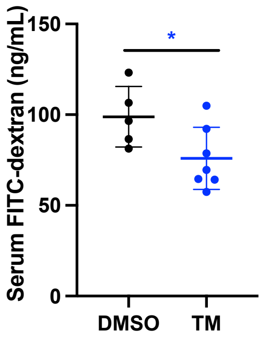

Figure S2. Sirt2 inhibition in mice showed reduced permeability to FITC-dextran. *$p < 0.05$ (Student's t test). Error bars: ± SD.

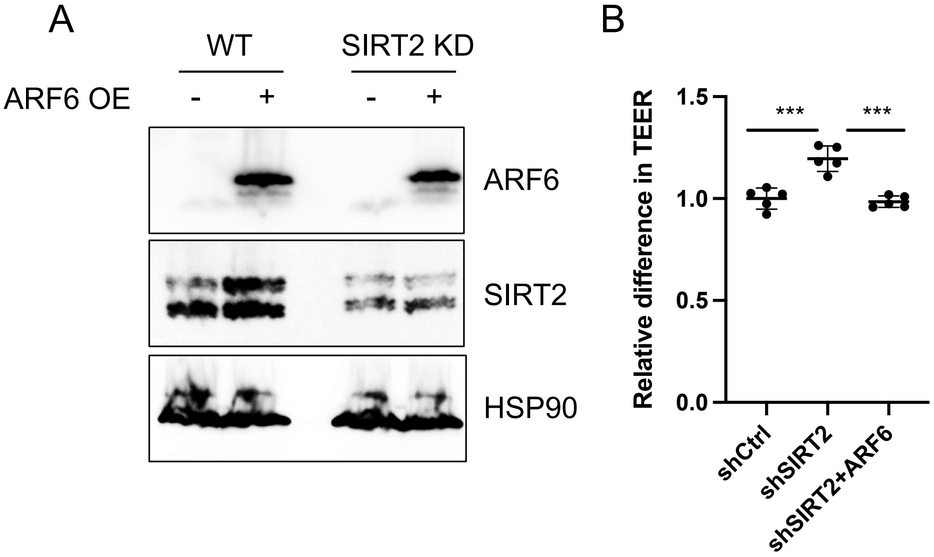

Figure S3. ARF6 overexpression rescued the effect of SIRT2 inhibition on epithelial barrier integrity. (A) Immunoblots of ARF6 and SIRT2 in WT and SIRT2 knockdown Caco2 cells. (A) TEER assay in WT, SIRT2 knockdown, and SIRT2 knockdown with ARF6 expressed Caco2 cells.

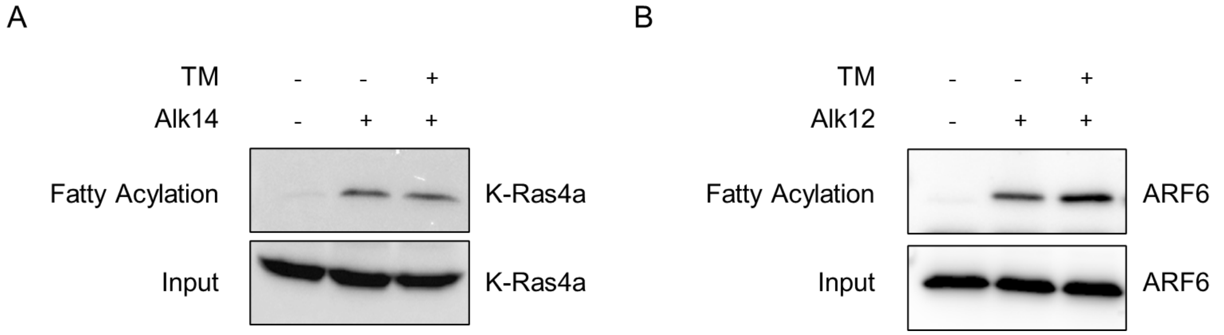

Figure S4. SIRT2 inhibitor's effect is substrate dependent. (A) The fatty acylation level of ARF6 in Caco2 cells treated with DMSO or 25 μM TM for 24 h, and with 50 μM Alk14 for 6 h. (B) The fatty acylation level of K-Ras4a in Caco2 cells treated with DMSO or 25 μM TM for 24 h, and with 50 μM Alk12 for 6 h. The fatty acylation level was detected using Alk12 or Alk14 labeling.
